# Critical Role of Transcription Factor PU.1 in the Function of the OX40L/TNFSF4 Promoter in Dendritic Cells

**DOI:** 10.1038/srep34825

**Published:** 2016-10-06

**Authors:** Takuya Yashiro, Mutsuko Hara, Hideoki Ogawa, Ko Okumura, Chiharu Nishiyama

**Affiliations:** 1 Laboratory of Molecular Biology and Immunology, Department of Biological Science and Technology, Faculty of Industrial Science and Technology, Tokyo University of Science, 6-3-1 Niijuku, Katsushika-ku, Tokyo, 125-8585, Japan; 2 Atopy Research Center, Juntendo University School of Medicine, 2-1-1 Hongo, Bunkyo-ku, Tokyo, 113-8421, Japan; 3 Research Fellow of Japan Society for the Promotion of Science, 5-3-1 Koujimachi, Chiyoda-ku, Tokyo 102-0083.

## Abstract

PU.1 is a hematopoietic lineage-specific transcription factor belonging to the Ets family. We investigated the role of PU.1 in the expression of OX40L in dendritic cells (DCs), because the regulatory mechanism of cell type-specific expression of OX40L, which is mainly restricted to antigen-presenting cells, is largely unknown despite the critical involvement in Th2 and Tfh development. PU.1 knockdown decreased the expression of OX40L in mouse DCs. Chromatin immunoprecipitation (ChIP) assays demonstrated that PU.1 constitutively bound to the proximal region of the OX40L promoter. Reporter assays and electrophoretic mobility shift assays revealed that PU.1 transactivated the OX40L promoter through direct binding to the most-proximal Ets motif. We found that this Ets motif is conserved between mouse and human, and that PU.1 bound to the human OX40L promoter in ChIP assay using human monocyte-derived DCs. ChIP assays based on ChIP-seq datasets revealed that PU.1 binds to several sites distant from the transcription start site on the OX40L gene in addition to the most-proximal site in mouse DCs. In the present study, the structure of the OX40L promoter regulated by PU.1 is determined. It is also suggested that PU.1 is involved in mouse OX40L expression via multiple binding sites on the gene.

Dendritic cells (DCs) are professional antigen-presenting cells (APCs) that reside in peripheral tissue and survey the body for pathogens. When immature DCs recognize microbial structures such as pathogen-associated molecular patterns (PAMPs) using pattern recognition receptors, they develop into mature DCs with up-regulation of MHC and costimulatory molecules. The OX40 ligand (OX40L, also known as TNFSF4 or CD252) is a costimulatory molecules that is mainly expressed on APCs, including mature DCs, B cells, and macrophages^1^. OX40L interacts with OX40 (also CD134) that is preferentially expressed on activated CD4 T cells. The OX40-OX40L interaction plays a key role in the control of the helper T-cell-mediated immunity at multiple points, including Th priming, effector cell function, and the generation and maintenance of memory^2^,^3^,^4^,^5^. This pathway is particularly important for the generation of Th2 responses both *in vitro* and *in vivo*^2^,^6^,^7^,^8^,^9^. Furthermore, activation of the OX40 signal strongly suppresses the TGF-β-induced conversion of naïve T cells into Foxp3^+^ regulatory T (Treg) cells^10^,^11^. In fact, knockout mice lacking the OX40 or OX40L gene products exhibited impaired T-cell function and subsequently ameliorated clinical symptoms in T-cell-mediated diseases such as asthma, inflammatory bowel disease, contact hypersensitivity (CHS), and experimental autoimmune encephalomyelitis^12^,^13^,^14^,^15^,^16^. In contrast, OX40L-transgenic mice developed a greater severity of these immune disorders^17^.

Despite the importance of OX40L in immune responses, the mechanism of the transcriptional regulation of OX40L gene remains to be elucidated. Previous studies showed that OX40L expression in DCs is up-regulated by several stimulatory signalings, including thymic stromal lymphopoietin (TSLP), CD40, PAMPs, and IFN-α^18^,^19^,^20^,^21^. TSLP, an epithelial cell-derived IL-7-like cytokine, up-regulates the expression of OX40L in DCs, and the TSLP-activated DCs induce inflammatory Th2 responses through OX40L^21^,^22^. In addition, it was recently demonstrated that TSLP induces OX40L expression in DCs through the binding of NF-κB p50 and RelB to the OX40L promoter^23^. On the other hand, vitamin D treatment enhanced the binding of vitamin D receptor to the OX40L promoter and in turn suppressed the binding of NF-κB p50 to the promoter, resulting in a reduction in OX40L promoter activity^24^. Polymorphisms in the *OX40L* promoter are linked to the susceptibility to systemic lupus erythematosus (SLE) and myocardial infarction^25^,^26^, suggesting that the strength of the *OX40L* promoter is associated with immune-related diseases.

PU.1 is a hematopoietic lineage-specific transcription factor that belongs to the Ets family. It has been proposed that graded levels of PU.1 expression by hematopoietic progenitors are determinative of their lineage commitment because high levels of PU.1 direct macrophage differentiation and low levels are sufficient for fetal B cell development^27^,^28^, whereas intermediate levels of PU.1 were required for granulocytes^29^. Analysis of PU.1/GFP reporter mice showed that PU.1 was expressed in all DC subsets, with myeloid DCs expressing a characteristic high amount of PU.1 and plasmacytoid DCs expressing a low level^30^. Several studies, including ours, have demonstrated that PU.1 transactivates the genes of DC-characteristic molecules, such as CIITA, CD80, CD86, TNF-α and IL-12 p40^31^,^32^,^33^. PU.1 regulates gene expression by binding to canonical Ets motifs not only as a monomer but also as a heterodimer with interferon regulatory factor 4 (IRF4) or IRF8, alternatively forming a complex with several transcription factors, including C/EBPα and β, and c-Jun^34^.

In this study, we investigated whether PU.1 regulates the expression of OX40L in DCs. We found that PU.1 binds to the Ets motif located in the 5′-flanking region proximal to the transcriptional start site and transactivates the OX40L gene both in mouse and human DCs.

## Results

### Effects of PU.1 knockdown on the mouse OX40L expression

**Figure 1:**
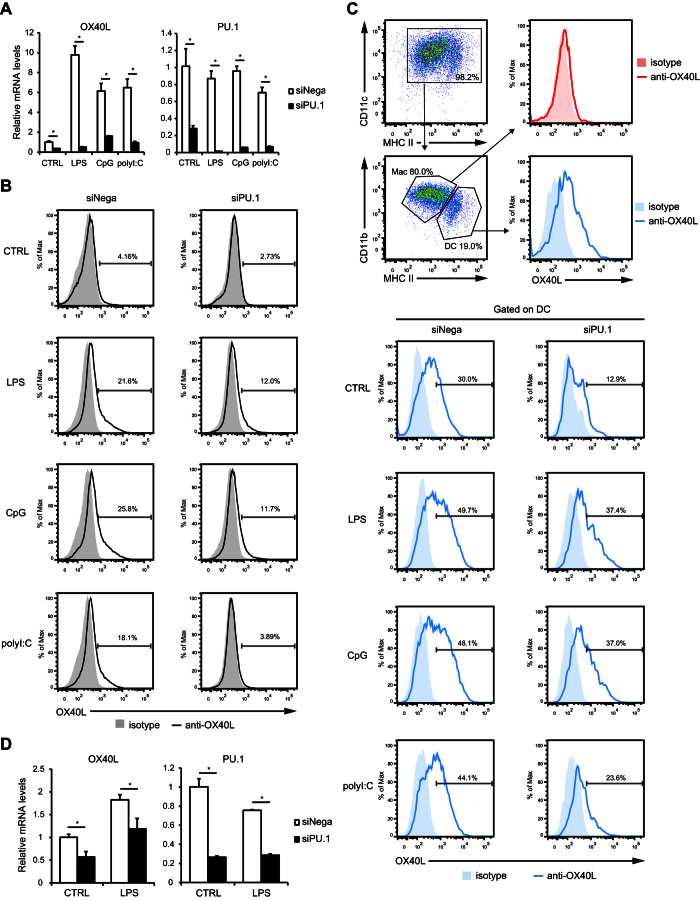
Effects of PU.1 knockdown on OX40L expression in mouse DCs. BMDCs were transfected with either negative control siRNA (siNega) or PU.1 siRNA (siPU.1). At 32 h after transfection, the cells were left untreated or stimulated with 1 μg/ml LPS, 1 μg/ml CpG, or 50 μg/ml poly I:C for 16 h (mRNA) or 40 h (flow cytometry). (**A**) Relative mRNA levels were determined by quantitative RT-PCR after normalizing to mouse GAPDH mRNA levels. Data are expressed as the ratio of the expression level of the respective negative control siRNA-transfected cells without stimulation. Results are shown as means ± S.D.s (*n* = 3). (**B**) Cells were stained with a PE-conjugated OX40L antibody. Cell-surface fluorescence was analyzed using a flow cytometer. Similar results were obtained in three separate experiments. (**C**) Surface expression level of OX40L on DC type cells and macrophage type cells in BMDCs. (**D)** mRNA levels of OX40L and PU.1 in PU.1 siRNA-introduced splenic DCs. Similar results were obtained in two independent experiments (C and D). *p < 0.05.

To evaluate the effect of PU.1 suppression on OX40L expression, BMDCs were transfected with PU.1 small interfering RNA (siRNA) and stimulated with potent activators of DCs such as LPS (a ligand for TLR4), CpG (for TLR9), and poly I:C (for TLR3). We observed approximately 6- to 10-fold increases in OX40L mRNA levels after TLR ligand-induced maturation of bone marrow-derived DCs (BMDCs) (open bars in Fig. 1A left). OX40L mRNA levels decreased significantly in both immature and mature BMDCs upon knockdown of PU.1 (Fig. 1A). Then, we examined whether PU.1 knockdown affected the protein levels of OX40L. Flow cytometric analysis using a PE-conjugated anti-OX40L Ab showed that OX40L was barely present on the cell surface of immature BMDCs but was clearly detected in mature BMDCs (Fig. 1B left). We confirmed that PU.1 knockdown led to a marked decrease in OX40L protein levels in both immature and mature BMDCs (Fig. 1B). These results suggest that PU.1 is involved in the expression of OX40L in BMDCs.

Recently, Helft *et al*. reported a heterogeneous population of BMDCs generated by GM-CSF culture system^35^. Briefly, GM-CSF-derived CD11c^+^/MHCII^+^ BMDCs are categorized into two cell types, one is CD11c^+^/MHCII^hi^ with DC phenotype and another is CD11c^+^/MHCII^int^ with macrophage phenotype. To examine the expression level of OX40L in each cell type, we performed flow cytometric analysis with using the same markers (CD11c, MHCII, and CD11b) as used in the previous study^35^ and found that DC type cells expressed higher amount of OX40L than macrophage type cells (Fig. 1C top). TLR ligand-mediated stimulation dramatically up-regulated OX40L expression on DCs. When PU.1 siRNA was introduced, the expression levels of OX40L on both non-stimulated DCs and stimulated DCs were markedly suppressed (Fig. 1C bottom). Taken together, these results demonstrate that DC type population rather than macrophage type population expresses higher amount of OX40L, which is down-regulated by PU.1 knockdown.

We also evaluated the effect of PU.1 knockdown on OX40L expression in primary cells with using DCs freshly isolated from mouse spleen. As shown in Fig. 1D, PU.1 mRNA level was markedly decreased in PU.1 siRNA-introduced splenic DCs (right). In this experimental condition, OX40L mRNA level, which was approximately 2-fold increased by LPS stimulation, was significantly reduced in both immature and mature DCs (left). From this result, it was confirmed that the involvement of PU.1 in OX40L expression is observed not only in *in vitro* generated DCs but also in primary DCs.

### Endogenous PU.1 binds to the proximal region of the mouse OX40L promoter

**Figure 2:**
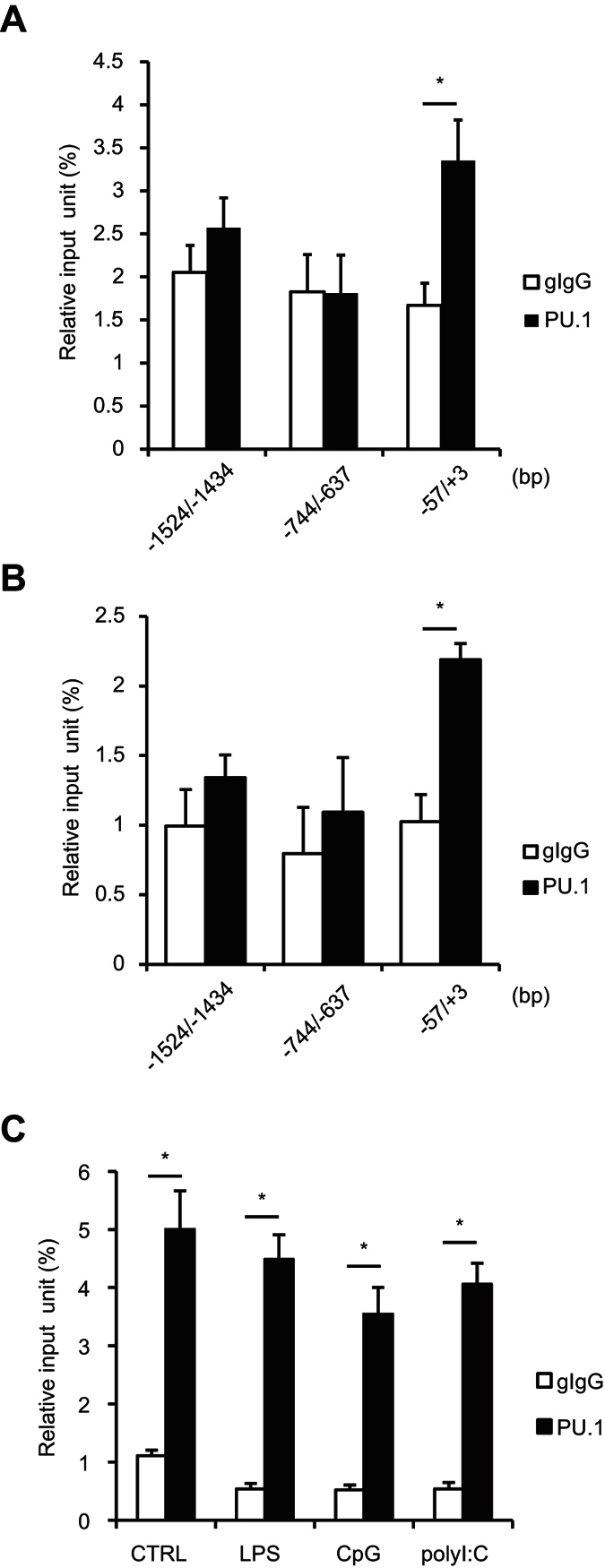
Analysis of PU.1-binding region in the mouse OX40L promoter. BMDCs without stimulation (**A**) freshly prepared splenic DCs (**B**) and BMDCs stimulated with indicated reagents for 16 h (**C**). ChIP assay was performed by using either goat IgG (gIgG) or anti-PU.1 Ab (PU.1). The amounts of immunoprecipitated chromatin were determined by quantitative PCR amplifying the indicated region of the OX40L promoter. Data are expressed as percentage of the input for each ChIP assay. Results are means ± S.D.s (*n* = *3*). Similar results were obtained in three independent experiments. *p < 0.05.

To investigate whether PU.1 binds to the endogenous OX40L promoter in chromosomal DNA, we initially performed chromatin immunoprecipitation (ChIP) assays using BMDCs (Fig. 2A) and splenic DCs (Fig. 2B). A markedly higher amount of DNA was immunoprecipitated by the anti-PU.1 Ab in compared with that of a control Ab when a primer set to amplify the region −57/+3 (the most proximal to the transcriptional initiation site) was used to detect DNA, whereas significant binding of PU.1 was not detected in ChIP assays targeting other upstream regions −1524/−1434 and −744/−637 (Fig. 2A and B). We next examined the effect of TLR ligand-induced maturation on the level of PU.1 binding to the OX40L promoter. The amount of DNA (around −57/+3) immunoprecipitated with the anti-PU.1 Ab in TLR ligand-treated BMDCs was similar to that in non-treated BMDCs (Fig. 2C), even though TLR-mediated stimulation significantly up-regulated the OX40L expression, as shown in Fig. 1. These results indicate that PU.1 specifically binds to the proximal region of the mouse OX40L promoter and the PU.1-binding is constitutive, which is not affected by the TLR ligand-dependent maturation of DCs.

### Identification of the OX40L promoter region necessary for transcriptional activity and for response to PU.1-mediated transactivation

In experiments by using mouse dendritic cell line, JAWS II, we obtained similar results to those of BMDCs. Briefly, the mRNA level of OX40L was decreased by siRNA-mediated PU.1 knockdown (Supplementary Fig. 1A), and PU.1 bound to the proximal region of the mouse OX40L promoter (Supplementary Fig. 1B), suggesting that PU.1 positively regulates the gene expression of OX40L with recruitment to the promoter in JAWS II cells as well.

**Figure 3:**
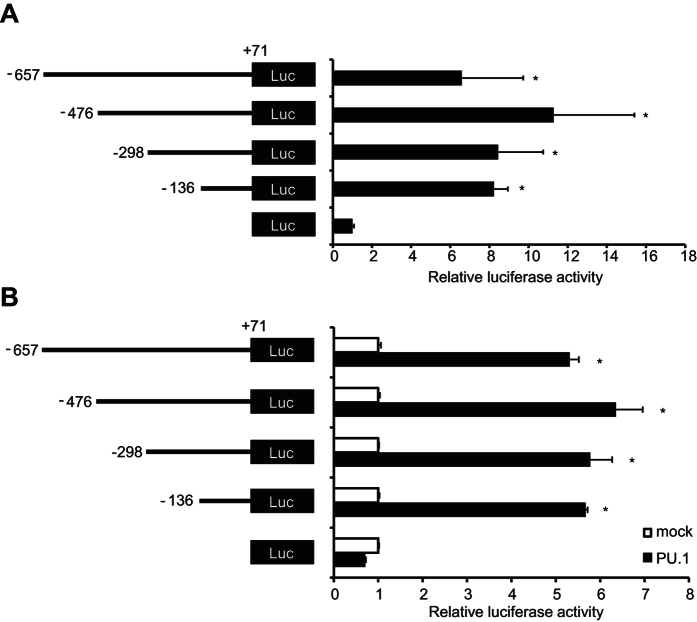
Analysis of *cis*-element in the mouse OX40L promoter. (**A**) JAWSII cells were transfected with reporter plasmids for renilla luciferase as an internal control and with reporter plasmids for firefly luciferase containing various lengths of the mouse OX40L promoter. (**B**) 293T cells were transfected with either empty (mock) or PU.1-expression (PU.1) plasmids together with the reporter plasmids as in (**A**). At 48 h after transfection, dual-luciferase assay was performed. Firefly luciferase activities were normalized to renilla luciferase activities. Data are expressed as the ratio of the luciferase activity of the respective promoter-less plasmid-transfected cells. All results are shown as means ± S.D.s (*n* = *3*). Similar results were obtained in three independent experiments. *p < 0.05 versus promoter-less reporter plasmid (**A**) or versus mock transfectant (**B**).

Therefore, we performed luciferase assays using JAWS II cells in order to evaluate the activity of the OX40L promoter. When JAWS II cells were transfected with a series of reporter vectors carrying various lengths of the mouse OX40L 5′-flanking regions, we found that the luciferase activities were enhanced as long as reporter genes contained the segment from −136 to +71 bp (Fig. 3A). This result indicated that *cis*-element(s) were located between −136 and +71. To assess the effect of PU.1 on OX40L promoter activity, we carried out luciferase assays using the human embryonic kidney (HEK293T) cells, which are useful for evaluation of the transactivation activity of hematopoietic cell-specific transcription factors. Luciferase activity driven by the shortest promoter (−136/+71) was significantly augmented with ectopic PU.1 to a similar extent as those driven by the reporter plasmids carrying longer promoter regions (Fig. 3B). These results suggest that PU.1-responsive *cis*-element(s) reside within the −136/+71 region, which is functional as a promoter in OX40L-expressing cells.

### PU.1 can bind directly to the most-proximal Ets motif, which is essential for the transcriptional function of the mouse OX40L promoter

**Figure 4:**
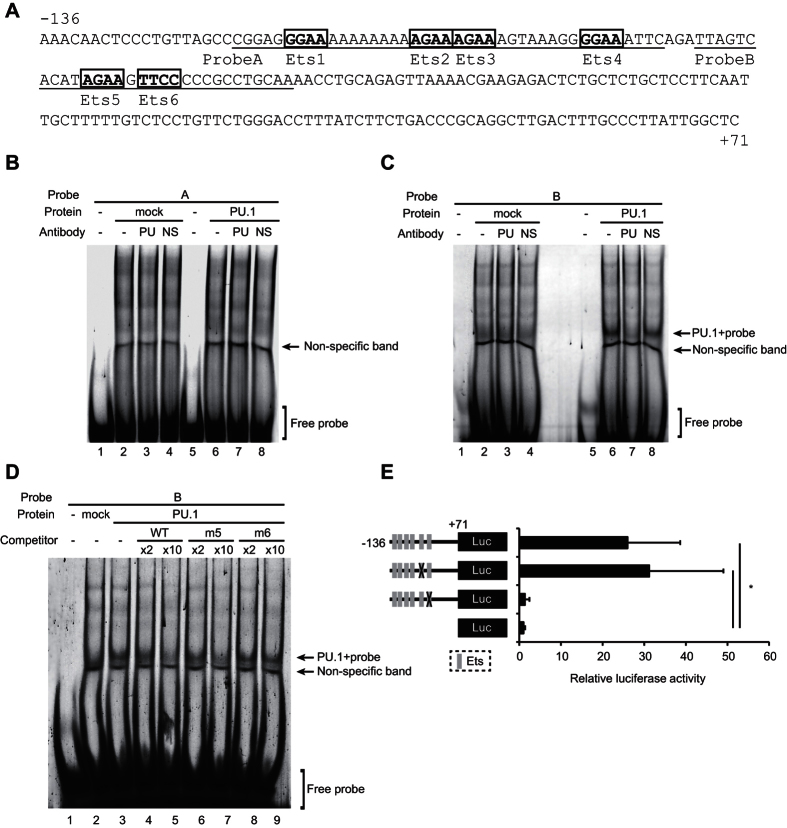
Identification of the PU.1-binding site. (**A**) Sequences of the −136/+71 region of the mouse OX40L promoter. Six Ets motifs, designated Ets1, 2, 3, 4, 5, and 6 are indicated in bold. Probes used in EMSA are underlined. (**B**–**D**) Nuclear extracts were prepared from 293T cells transfected with either empty (mock) or PU.1-expression plasmids (PU.1). The FLO-labeled probe A (**B**) or B (**C)** were incubated with the nuclear extracts in the presence of either anti-PU.1 (PU) or non-specific (NS) Abs. The FLO-labeled probe B was incubated with the nuclear extracts in the presence of 2-fold (×2) or 10-fold (×10) amounts of non-labeled WT or mutated competitor B (**D**). After electrophoresis in 5% acrylamide gels, fluorescence was detected. (**E**) JAWSII cells were transfected with reporter plasmids of WT or mutant promoters lacking Ets-motif(s) at the indicated sites. Data are expressed as in Fig. 3.

As shown in Fig. 4A, six putative PU.1 binding sequences (GGAA or AGAA) exist in the −136/+71 region of the mouse *OX40L* promoter. We termed these motifs Ets 1–6, respectively. To examine whether these Ets motifs are recognized by PU.1, electrophoretic mobility shift assay (EMSAs) were performed using the two probes underlined in Fig. 4A. No specific bands were detected, when nuclear extracts of 293T cells transfected with PU.1 expression vector was mixed with a fluorescein (FLO)-labeled probe A (Fig. 4B). When FLO-labeled probe B containing two motifs, Ets 5 and Ets 6, was mixed with the nuclear extract, a specific band appeared on an electrophoretic gel, which disappeared in the presence of an anti-PU.1 Ab but not a non-specific Ab (Fig. 4C, *lanes 5–8*). Next, we performed EMSAs with competitive double-stranded oligonucleotides to further identify the PU.1-binding site in this region. The shifted band completely disappeared upon the addition of an excess amount of the wild-type (WT) competitor or Ets 5-mutated competitor but was only slightly diminished by a mutant competitor lacking Ets 6 (Fig. 4D). These results clearly demonstrate that PU.1 is capable of binding to this −58/−55 TTCC sequence in the mouse *OX40L* promoter. To determine the involvement of these motifs in mouse OX40L promoter activity, luciferase assays using WT and mutant plasmids, in which nucleotide replacements were introduced at Ets 5 or Ets 6, were carried out using JAWS II cells (Fig. 4E). As expected from the EMSA results, mutation of Ets 6 significantly reduced promoter activity to the basal level. In contrast, luciferase activity driven by the mutant promoter lacking Ets 5 was similar to that of the WT. This result indicates that Ets 6 is critical for the mouse OX40L promoter function.

### Binding of PU.1 to the human *OX40L* promoter

**Figure 5:**
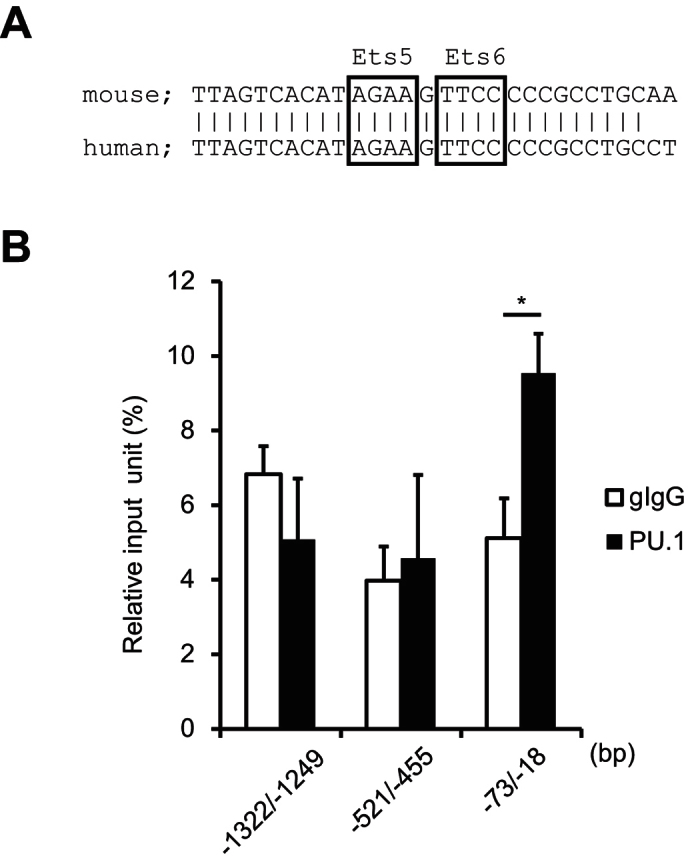
Effects of PU.1 in the expression of OX40L in human DCs. (**A**) Comparison of OX40L promoter sequence between mouse and human. (**B**) ChIP assays were performed using either goat IgG (gIgG) or anti-PU.1 Ab (PU.1). The amounts of immunoprecipitated chromatin were determined by quantitative PCR amplifying the indicated region of the human OX40L promoter. Data are expressed as percentage of the input for each ChIP assay. Results are shown as means ± S.D.s (*n* = 3). Similar results were obtained in three independent experiments. *p < 0.05.

In the alignment of nucleotide sequences of the mouse and human *OX40L* promoters (Fig. 5A), we found that the sequences of the identified PU.1 binding sites and the surrounding nucleotides in the mouse promoter were highly conserved with that in human. Then, to confirm the involvement of this region in the binding of PU.1 to the *OX40L* promoter in human DCs, we performed ChIP assays using human monocyte-derived DCs generated from CD14^+^ monocytes by culturing with human GM-CSF and IL-4. The amount of chromosomal DNA immunoprecipitated with the anti-PU.1 Ab was significantly higher than that with the control Ab (Fig. 5B), when a primer set amplifying the region containing the most proximal Ets motif was used for PCR, whereas a significant difference was not detected between the anti-PU.1 Ab and the control Ab in a more upstream region of the human *OX40L* promoter (Fig. 5B). This result suggests that PU.1 is also recruited to the proximal region of the *OX40L* promoter in human DCs.

### PU.1-binding regions distant from the transcription start site

**Figure 6:**
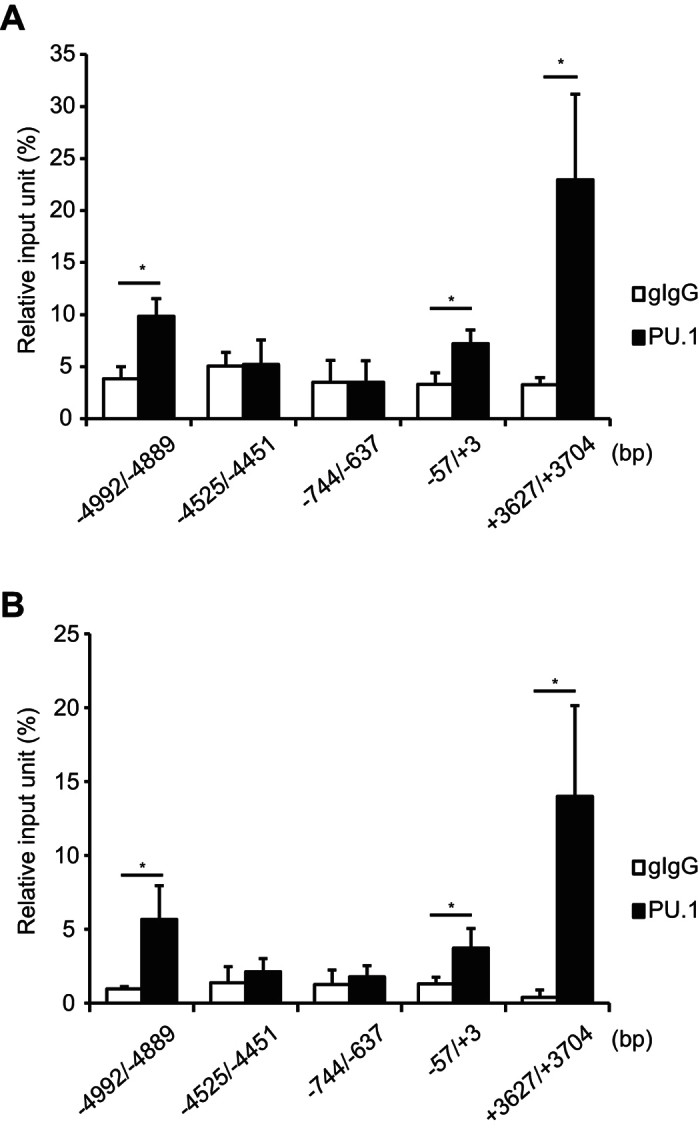
PU.1-binding regions distant from the transcription start site. ChIP assay was performed with BMDCs (**A**) and splenic DCs (**B**) by using either goat IgG (gIgG) or anti-PU.1 Ab (PU.1). Data are expressed as percentage of the input for each ChIP assay. Results are means ± S.D.s (*n* = *3*). Similar results were obtained in two independent experiments. *p < 0.05.

Although we identified the sequence TTCC at −58/−55 as a *cis*-enhancing element for PU.1-mediated transactivation of the promoter by reporter assays using a series of reporter plasmids carrying −657/+71 and its truncated variants, there is still a possibility that the *OX40L* gene possesses other PU.1-binding sites outside of the minimum promoter, because recent studies showed that PU.1 binds to the target genes through multiple sites^36^,^37^. Then, we searched PU.1-binding sites on the *OX40L* gene in mouse DCs by using published ChIP-seq data (NCBI GEO series GSE64767 and GSE57563, and on UCSC genome browser track data hub, http://www.molcell.rwth-aachen.de/dc/,^37^), and found several PU.1 peaks on the *OX40L* gene in addition to −58/−55 region. To evaluate the specific binding of PU.1 to the putative sites in our experimental condition, the amount of chromosomal DNA immunoprecipitated with anti-PU.1 Ab was determined by using additional primer sets. As shown in Fig. 6, further enriched binding of PU.1 was detected at −4.9 kb and +3.7 kb in BMDCs (A) and in splenic cDCs (B) under the conditions that specific binding was detected at −57/+3 but not at other *cis*-control sites (−4525/−4451 and −744/−637). These results indicate that PU.1 binds to the *OX40L* gene via multiple sites in mouse DCs.

## Discussion

We and others have demonstrated that PU.1 plays critical roles in DC functions. Briefly, PU.1 positively regulates the expression of CIITA, which is mainly involved in the transcriptional regulation of MHC class II^32^. Moreover, PU.1 transactivates the *CD80* and *CD86* genes, which provide co-stimulatory signals by engaging CD28 or CTLA-4 on T cells^31^. These findings indicated that PU.1 in DCs is required for the precise T cell stimulation, proliferation, and polarization. In the present study, we investigated whether PU.1 regulates the gene expression of OX40L, one of the co-stimulatory molecules predominantly involved in promoting the conversion from naïve T cells to Th2 cells and Tfh^38^,^39^. Although the functions of OX40L in DCs have been explored for the last several decades, the regulatory mechanism of OX40L gene expression is largely unknown. Here, we clearly demonstrated that PU.1 is a transcriptional activator of the OX40L gene in DCs.

The siRNA-mediated PU.1 knockdown significantly decreased the expression of OX40L in both of immature and mature DCs (Fig. 1). Additionally, ChIP assays using the anti-PU.1 Ab revealed that PU.1 bound to the proximal region of the OX40L promoter in a stimulation state-independent manner (Fig. 2C). These results suggested that PU.1 plays a role as an activator of basal transcription of the OX40L gene in BMDCs throughout immature and mature states. Although TLR ligand-dependent maturation increased the expression of OX40L, the amount of PU.1-binding to the proximal region of the OX40L promoter was not changed. Because TSLP induces OX40L expression through the binding of NF-κB p50 and RelB to the promoter^23^, the up-regulation of OX40L by the TLR ligand is induced by the activation of NF-κB, in contrast to the constitutive requirement of PU.1 for OX40L expression. Interestingly, NF-κB binding sites reported in the previous study^23^ is conserved between human and mouse, and closely locates to the Ets site identified in the present study. The OX40L promoter containing the *cis*-elements for PU.1-mediated basal transcription and NF-κB-mediated up-regulation might be a useful tool to reveal the cross-talk between PU.1 and NF-κB. It has been reported that IL-33 enhanced the OX40L expression in DCs and induced Th2 responses^40^. We also found that mIL-33 did not affect PU.1-binding to the proximal region of OX40L promoter, even when mIL-33 significantly up-regulated the expression of OX40L (data not shown), supporting the role of PU.1 in the constitutive expression of OX40L.

We showed that PU.1 specifically bound to −58/−55 TTCC of the mouse OX40L promoter by EMSA (Fig. 4). As shown in Fig. 5A, nucleotide sequences around this site are well conserved between human and mouse, suggesting that the PU.1-OX40L axis may be common in mammals. Indeed, ChIP assays revealed that PU.1 also bound to the proximal region of the human OX40L promoter in monocyte-derived DCs (Fig. 5).

Finally, we searched other PU.1-binding peaks outside of the minimum promoter on the OX40L gene in mouse DCs by using published ChIP-seq datasets and found the presence of several peaks. As expected from the datasets, further enriched binding of PU.1 was detected at these putative regions in our experimental condition using BMDCs and splenic DCs. In contrast to the high homology between mouse and human at just upstream of the transcription start site, the candidate sites possessing similar sequences as identified at −4.9 kb and +3.7 kb on the mouse OX40L gene were not identified in human gene so far. Furthermore, enriched PU.1-binding peak was not found on the OX40L gene in published ChIP-seq data using human CD14^+^ monocytes and monocyte-derived macrophages (UCSC Genome Browser on Human GRCh39/hg19 Assembly). Involvement of PU.1 in OX40L expression through multiple sites might be observed in mouse gene but not human gene.

Besides mature DCs, B cells and macrophages express OX40L^41^. Considering that PU.1 is expressed in these lineages, it is possible that the transcription of OX40L gene in all of these APCs is commonly regulated by PU.1. Of note, IgE-Ag complex-activated mast cells up-regulate the expression of OX40L, and interactions between OX40L on mast cells and OX40 on T cells is required for T cell proliferation^42^. Furthermore, Notch signaling confers antigen-presenting functions via up-regulation of OX40L expression on mast cells, which preferentially induce the differentiation of Th2^43^. In our previous studies, we detected the expression of PU.1 in mast cells, which was involved in the expression of FcεRI^44^ and CIITA^45^.

*In vivo* blockade of OX40-OX40L interactions resulted in a marked reduction in the infiltration of effector T cells into sites of inflammation and, therefore, in reduced clinical symptoms in several T cell-mediated diseases^46^,^47^. Furthermore, in mouse models of transplantation, blockade of both CD28 and OX40 pathways was more effective in preventing graft rejection than blockade of either alone^48^. Targeting the T cell costimulatory molecules CD28 and OX40 is thought to be promising strategy for controlling T cell-mediated immune disorders. We previously reported that PU.1 regulates gene expression of both CD80 and CD86 by binding directly to these promoters in DCs, and subcutaneous injection of PU.1 siRNA significantly suppressed CHS in a mouse model^31^. We found that a reduction in CHS by PU.1 siRNA was slightly greater than that by the combination of siRNAs for CD80 and CD86. The suppression of OX40L expression may contribute to the effect of PU.1 siRNA on this symptom of CHS. Considering that T cell-stimulating factor such as CD80, CD86, and OX40L are positively regulated by PU.1 in DCs, PU.1 knockdown may be a favorable strategy in some immune disorders.

## Materials and Methods

### Mice

BALB/c and C57BL/6 mice were purchased from Japan SLC (Hamamatsu, Japan). All animal experiments were performed in accordance with the approved guidelines of the Institutional Review Board of Juntendo University School of Medicine, Tokyo, Japan, and of Tokyo University of Science, Tokyo, Japan.

### Cells and reagents

BMDCs were generated from the femoral and tibial bone marrow cells of female mice as described previously^49^. Cells were incubated in RPMI 1640 (Sigma-Aldrich, St Louis, MO) supplemented with 10% heat-inactivated fetal calf serum, 100 U/mL of penicillin, 100 μg/mL streptomycin, 100 μM 2-mercaptoethanol, 10 μM Minimum Essential Medium nonessential amino acid solution, and 20 ng/mL of murine GM-CSF (PeproTech, London, United Kingdom) for 10 days. CD11c^+^ cells were isolated by using the MACS separation system with anti-mouse CD11c MicroBeads (#130-052-001) and an autoMACS (all from Miltenyi Biotech, Tubingen, Germany). Splenic DCs were also isolated from mouse spleen by using the MACS separation system with anti-CD11c MicroBeas^50^.

Mouse DC line JAWSII (CRL-11904, American Type Culture Collection, Manassas, VA) were maintained in MEM-α (GIBCO) supplemented with 20% FCS, 4 mM L-glutamine, 1 mM sodium pyruvate, 100 U/mL of penicillin, 100 μg/mL streptomycin, and 5 ng/mL of murine GM-CSF. All cells were incubated at 37 °C in a humidified atmosphere in the presence of 5% CO_2_.

LPS (#L3024) and poly I:C (#P0913) were purchased from Sigma-Aldrich. CpG-ODN (#tlrl-1826) was obtained from InvivoGen (San Diego, CA).

### siRNA experiments

PU.1 siRNA (Stealth Select RNAi, Sfpi1-MSS247676) and control siRNA (Stealth Negative Control) were obtained from Invitrogen (Carlsbad, CA). A 5 μl of aliquot of 20 μmol/L siRNA was introduced into 1 × 10^7^ BMDCs using a Mouse Dendritic cell Nucleofector kit (Lonza, Basel, Switzerland) using Nucleofector II (Lonza) set at Y-001.

### Quantitative RT-PCR

Total RNA was extracted from BMDCs using an RNeasy Micro Kit (QIAGEN, Hilden, Germany) according to the manufacturer’s instructions. Complementary DNA was synthesized from 2 μg of total RNA and amplified using a ReverTra Ace qPCR RT kit (TOYOBO, Osaka, Japan). Quantitative real-time PCR was performed using an Applied Biosystems StepOne real-time PCR system. Relative mRNA levels were obtained after normalization to the GAPDH transcript. The TaqMan ID numbers for the genes analyzed in the present study are as follows: mOX40L, Mm0043721_m1; mPU.1, Mm00488142_m1; hPU.1, Hs02786711_m1; mGAPDH, 4352339E; hGAPDH, 4326317E. For detection of the human OX40L transcripts, the following primers were used with SYBR Green PCR Master Mix (#4309155, Applied Biosystems, forward, 5′-CTGTGCTTCACCTACATCT-3′, reverse, 5′-AGGATACCGATGTGATACC-3′.

### Flow cytometric analysis

A PE-conjugated anti-OX40L antibody (RM134L; eBioscience) was used to stain cell-surface OX40L after blocking Fc receptors with 2.4G2 (BD PharMingen). PerCP-conjugated anti-I-A/I-E (MHCII) (M5/114.15.2; BioLegend), PE Cy7-conjugated anti-CD11c (N418; TONBO bioscience), and APC Cy7-conjugated anti-CD11b (M1/70; TONBO bioscience) were used to characterize BMDCs. Cell-surface fluorescence was detected using MACSQuant (Miltenyi Biotech) and analyzed by Flowjo (Tomy digital biology, Tokyo, Japan).

### Luciferase assay

Various lengths of the 5′ flanking region of *OX40L* were amplified from mouse genomic DNA by using PCR and inserted into the multi-cloning site of pGL-4 Basic (Promega, Madison, WI) to generate reporter plasmids. Mutant reporter plasmids were generated by using a PrimeSTAR Mutagenesis basal kit (TaKaRa Bio, Shiga, Japan). The nucleotide sequences of the primers are listed in Supplemental Table 1. An expression plasmid for flag-tagged PU.1 was constructed by inserting the fragment encoding mouse PU.1 into the EcoRI-SalI site of a p3 × Flag-CMV-7.1 vector (SIGMA).

Cells of 293T were transfected with 500 ng reporter plasmid, 500 ng expression plasmid, and 10 ng pRL-null (Promega) using FuGENE HD (Promega). JAWSII cells were transfected with 1 μg reporter plasmid and 50 ng pRL-CMV (Promega). At 48 h after transfection, luciferase activity was determined with using Micro Lumat Plus (Berthold Technologies, Bad Wildbad, Germany) and a Dual-luciferase assay kit (Promega), as described previously^51^.

### EMSA

An EMSA was performed based on a method described previously^52^. Synthesized oligonucleotides of the target sequence and its complementary sequence, both of which were labeled with fluorescein (FLO) at the 5′-end, were annealed to prepare the double-stranded DNA probe. Nuclear proteins were extracted from 293T cells, which were transfected with either pCR3.1-PU.1 or pCR3.1-empty vector, by using NE-PER Nuclear and Cytoplasmic Extraction Reagents (Thermo Scientific, Rockford, IL). The band shift on a polyacrylamide gel was analyzed with Typhoon FLA 9500 (GE Healthcare).

### ChIP assay

ChIP assays were performed as described previously^51^. Anti-PU.1 (the same clone as that used for EMSA analysis) and goat IgG (#02-6202, Invitrogen) were used for immunoprecipitation. Quantitative PCR of precipitated chromosomal DNA was performed using an Applied Biosystems StepOne real-time PCR system. The nucleotide sequences of the primer sets used for quantitative PCR are described in Supplementary Table 2.

### Statistical analysis

Statistical analysis was performed using a two-tailed Student’s t-test with *p* values < 0.05 considered significant.

## Additional Information

**How to cite this article**: Yashiro, T. *et al*. Critical Role of Transcription Factor PU.1 in the Function of the OX40L/TNFSF4 Promoter in Dendritic cells. *Sci. Rep.* **6**, 34825; doi: 10.1038/srep34825 (2016).

## Supplementary Material

Supplementary Information
