# Supplementary material for: Critical Role of Transcription Factor PU.1 in the Function of the OX40L/TNFSF4 Promoter in Dendritic Cells
Source: Sci Rep. 2016 Oct 6;6:34825. doi: 10.1038/srep34825 (PMC5052589; doi:10.1038/srep34825)
Supplement: Supplementary Information [file srep34825-s1.pdf]

Critical Role of Transcription Factor PU.1 in the Function of the OX40L/TNFSF4 Promoter in Dendritic Cells

Takuya Yashiro, Mutsuko Hara, Hideoki Ogawa, Ko Okumura, and Chiharu Nishiyama

Department of Biological Science and Technology,

Faculty of Industrial Science and Technology, Tokyo University of Science,

6-3-1 Niijuku, Katsushika-ku, Tokyo, 125-8585, Japan

Atopy Research Center, Juntendo University School of Medicine,

2-1-1 Hongo, Bunkyo-ku, Tokyo, 113-8421, Japan

Supplementary Table 1. Sequence of primers used in construction of reporter vectors.

5’ position	Direction	Sequence
-657	Forward	ACACggtaccGGGTGGGGAATGACAGAATT
-476	Forward	TGATggtaccTGTAACTATAAGACCCCTTC
-291	Forward	ACACggtaccGGTTTAAGACATCTTGAGGG
-136	Forward	ACACggtaccAAACAACTCCCTGTTAGCCC
+71	Reverse	CACCagatctGAGCCAATAAGGGCAAAGTC

Mutation site	Direction	Sequence
Ets5	Forward	GTCACTCTAGAGTTCCCCCGCCTGCAAA
	Reverse	GAACTCTAGAGTGACTAATCTGAATTTC
Ets6	Forward	AGAAGTCGACCCGCCTGCAAAACCTGC
	Reverse	GGCGGGTCGACTTCTATGTGACTAATCTGAATT

Supplementary Table 2. Sequence of primers used in ChIP assay.

Species	Amplified region	Direction	Sequence
Mouse	-4992/-4889	Forward	TGGTGGGAAGAGATGAAAGACA
		Reverse	CTTACCTCCTTCTTTTTCTATTTCTGATAATC
	-4525/-4451	Forward	AACCCAGGAGCCACACCTT
		Reverse	AGTACTGGCTTTTCTTTAGCCTTG
	-1524/-1434	Forward	GCTGCAGAGTAGCCTATGTG
		Reverse	GAGGCGTTGCATTTGGAGAC
	-744/-637	Forward	AGAGCAACAAGCCTCAAATG
		Reverse	AATTCTGTCATTCCCCACCC
	-57/+3	Forward	TCCCCCGCCTGCAAA
		Reverse	AATTGAAGGAGCAGAGCAGAGTCT
	+3627/+3704	Forward	ACTTCTTGTCTCTTGTTTCTGTTAGGAA
		Reverse	ACATCACCAAGAGACTGAATTCTAACTC
Human	-1322/-1249	Forward	TTTTGGCTTGGAGTCTATGATATTGT
		Reverse	GAGAACATACTGGTGTTGAGAAATTTAAG
	-521/-455	Forward	TGCAATAGACTACAACCAAGGATCTC
		Reverse	GAAAAAGACAAAACAAGAAAATAGGACACT
	-73/-18	Forward	CCGCCTGCCTGCAAAA
		Reverse	GAAAGAGCAAAGCGGACTCTCT

*+1; Transcription start site

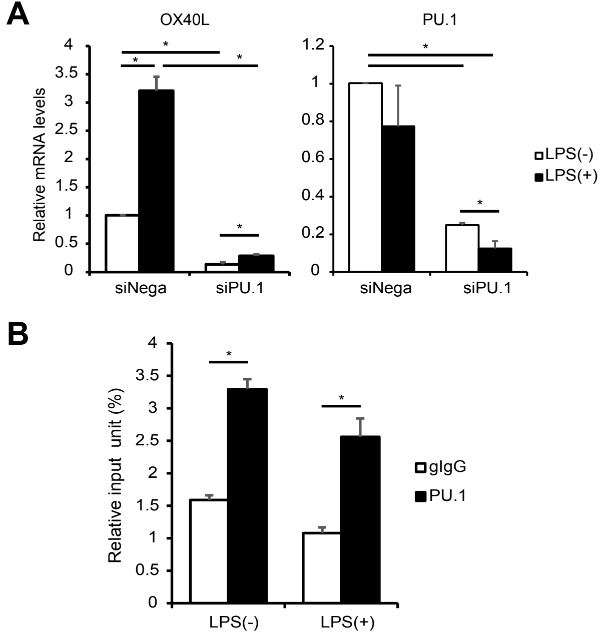

Supplemental Figure 1. Expression of OX40L in PU.1 siRNA-transfected JAWSII cells and specific binding of PU.1 to the OX40L promoter in JAWSII cells.

(A) JAWSII cells were transfected with either negative control siRNA (siNega) or PU.1 siRNA (siPU.1). At 32 h after transfection, the cells were left untreated (open bars) or stimulated (closed bars) with 1 μg/ml LPS for 16 h. Relative mRNA levels were determined by quantitative RT-PCR after normalizing to mouse GAPDH mRNA levels. Data are expressed as the ratio of the expression level of the respective control siRNA-transfected cells without stimulation. Results are shown as means ± S.D.s (*n*=3).

(B) ChIP assay was performed by using either goat IgG (gIgG) or anti-PU.1 Ab (PU.1). The amounts of immunoprecipitated chromatin were determined by quantitative PCR amplifying the indicated region of the OX40L promoter. Data are expressed as percentage of the input for each ChIP assay. Results are means ± S.D.s (*n=3*). Similar results were obtained in three independent experiments.
